# Antibody and Memory B Cell Responses in Hepatitis E Recovered Individuals, 1–30 Years Post Hepatitis E Virus Infection

**DOI:** 10.1038/s41598-019-40603-9

**Published:** 2019-03-11

**Authors:** Shruti P. Kulkarni, Meenal Sharma, Anuradha S. Tripathy

**Affiliations:** Hepatitis Group, Indian Council of Medical Research- National Institute of Virology, 130/1, Sus Road, Pashan, Pune, 411021 Maharashtra India

## Abstract

Generation and persistence of anti-hepatitis E virus (HEV) antibodies are synonymous with the development of immunity and considered as correlates of protection against HEV infection. However, issues like longevity of immunological memory following recovery from hepatitis E still remains a puzzle. It is critical to understand whether anamnestic response exists for protection from HEV re-infection. The levels and persistence of anti-HEV antibodies were assessed in hepatitis E recovered individuals 1–30 years post HEV infection. The frequencies and functionality of recombinant HEV capsid protein (rORF2p)-stimulated memory B and T cells were also investigated 1–16 years post infection. Anti-HEV antibodies persisted in 91% of hepatitis E recovered individuals. HEV-specific memory B cell responses were detected in 95% of seropositive hepatitis E recovered individuals. CD4^+^ and CD8^+^ T cells displayed an effector memory cell phenotype in hepatitis E recovered individuals. In conclusion, long-lived anti-HEV antibodies and HEV-specific memory B cells are maintained for several years in hepatitis E recovered individuals. Involvement of CD4^+^ and CD8^+^ effector memory T cells is an important observation since it is inextricably linked to long-lasting protective immunity. In addition to anti-HEV antibodies, possible role of memory B cell response against HEV re-infection could also be considered.

## Introduction

Hepatitis E, caused by hepatitis E virus (HEV) infection, is a disease of global public health concern with an annual estimate of 20 million cases of HEV infection, over 3.3 million symptomatic cases and 44,000 deaths^1^. Hepatitis E, mostly a self-limiting inflammatory liver disease, can progress to fulminant hepatic failure in pregnant women especially in the third trimester^2^, and may take a chronic course with serious clinical manifestations in HEV genotype 3 and 4 infected immunocompromised individuals. Hyperendemicity of HEV infection in India and higher incidence of subclinical infections make it difficult to say exactly when one seropositive individual had got the exposure. Thus, follow-up of individuals clinically recovered from HEV infection can provide information regarding immunological memory/protective response. More than three decades after the discovery of HEV, a question of paramount importance still remains unanswered: Will hepatitis E recovered individuals mount a protective immune response upon re-exposure to HEV? This issue can be addressed by the assessment of the three components of immunological memory namely, antibody, memory B and T cell responses in hepatitis E recovered individuals.

There are conflicting reports regarding the persistence and protective role of anti-HEV antibodies, the first line of defense against re-infection. Anti-HEV antibodies were reported to persist for 5 and 12 years post HEV infection in epidemic and sporadic settings respectively and were statistically estimated to persist for >50 years^3^. Absence of any cases of hepatitis E during follow-up pointed towards the protective role of pre-existing antibodies against re-infection^3^. Antibodies have thus conventionally been referred as immune correlates of protection against HEV infection. However, waning of antibodies with time was observed in a large proportion (~95%) of infected individuals^4^. Assessment of seropositivity in archived serum samples of blood donors showed that 5/23 donors turned seronegative over a period of 22 years^5^. A much higher rate (50%) of seroreversion was reported in baseline seropositive individuals that were followed up for 1–22 years^6^. Another study showed that anti-HEV antibodies decline after 5 years and more distinctly over time, albeit with a low rate of seronegativity^7^. Recent reports have shown the persistence of anti-HEV antibodies at least for 10 years post infection in 80% of the studied individuals^8^ and a seroreversion rate of 22.6% over a period of 12 years^9^.

In hepatitis A virus (HAV) and hepatitis B virus (HBV) infections, despite waning of antibodies overtime, functional memory B cells were detectable for several years imparting a life-long protective immunity^10,11^. Despite advances in understanding humoral immune responses, a big lacuna exists regarding memory B cell responses against HEV infection.

Memory T cell development was shown to be essential for controlling hepatitis C virus (HCV) re-infection^12^, and HCV-specific memory T cells were shown to persist for 18 years after spontaneous viral clearance in recovered individuals^13^. The presence of HEV-specific memory T cells was observed for more than 1.5 years post HEV genotype 3 infection upon recovery from clinical hepatitis E^14^. Another group reported persistence of functional memory T cells for over 10 years post HEV genotype 3 infection^15^.

It is largely unclear for how long HEV-specific anamnestic B and T cell responses exist and whether they have a role against re-infection. With this background, this study was designed to investigate the longevity of antibody, memory B and T cell responses in hepatitis E recovered individuals, 1–30 years post HEV infection.

## Results

### **C**haracteristics of study groups

The characteristics of the study groups are represented in Table 1.

**Table 1:** Clinical characteristics of study groups. Data are shown as median (range); NA: Not applicable.

Parameter	Acute hepatitis E patients	Hepatitis E recovered individuals	Healthy controls
Sample size	31	35	29
Sex ratio (Male:Female)	2:1	1:1	4:3
Age (years)	29 (11–64)	42 (12–80)	28 (21–58)
Anti-HEV IgM	Positive	Negative	Negative
Anti-HEV IgG	Positive	Positive (N = 32)/Negative (N = 3)	Negative
Post onset days of illness	6 (2–17) days	3 (1–30) years	NA

### **Anti-HEV IgG positivity in hep**atitis E recovered individuals

**Figure 1:**
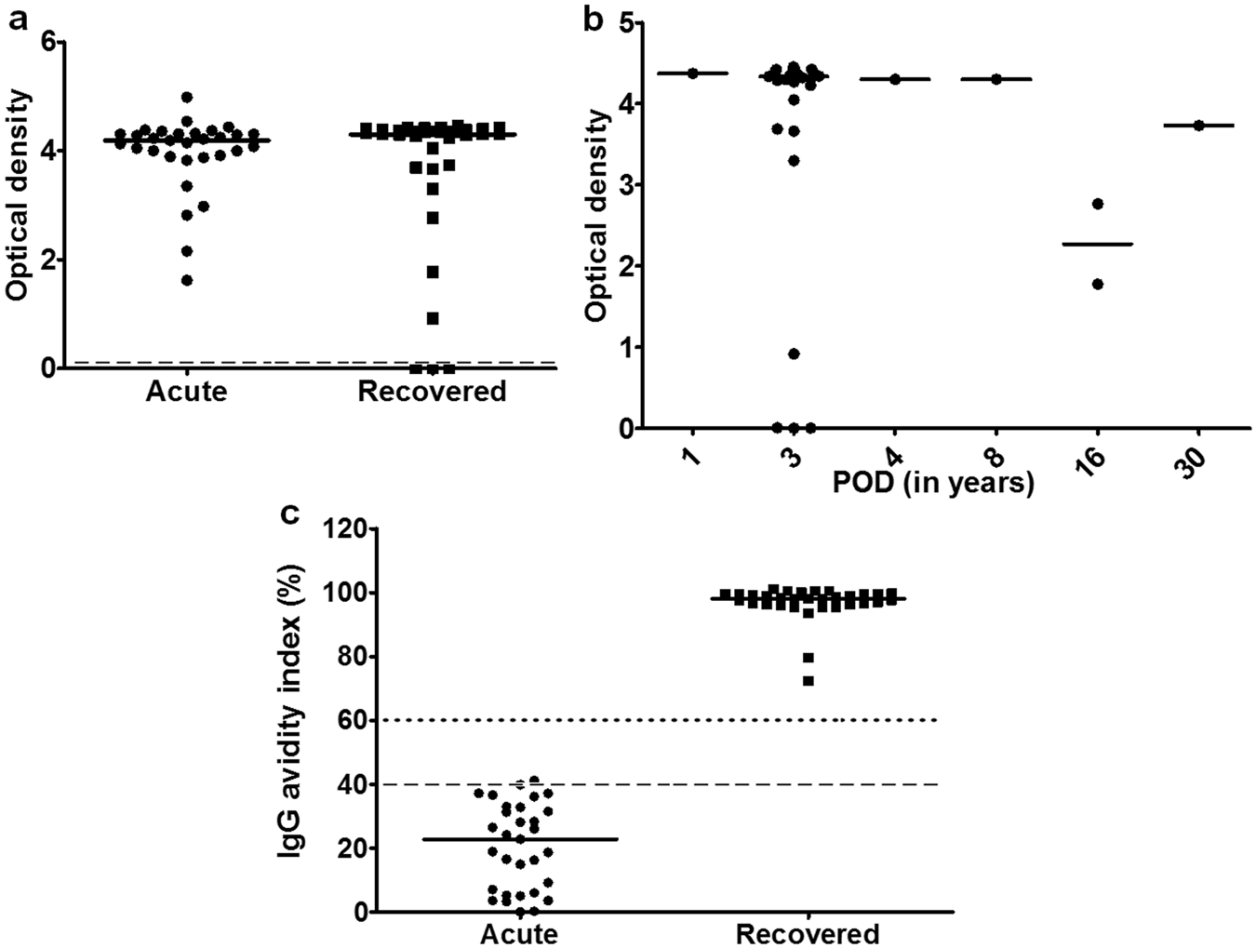
Anti-HEV IgG antibody positivity. Plasma of all study participants was used for detection of anti-HEV IgG antibodies. In positive samples, IgG avidity indices were determined. The dots represent individual values and bars represent median values. (**a**) Anti-HEV IgG positivity in acute hepatitis E patients (n = 31) and hepatitis E recovered individuals (n = 35). Dashed line shows the cut off value for the assay (**b**) Anti-HEV IgG antibodies in all hepatitis E recovered individuals 1–30 years post infection (**c**) IgG avidity indices of all acute hepatitis E patients and hepatitis E recovered individuals. An index <40% was considered as low, >60% as high and 40–60% as equivocal and are represented by the dotted and dashed lines.

All the acute hepatitis E patients were positive for anti-HEV IgG antibodies and in the recovered group, 32/35 (91%) individuals were anti-HEV IgG positive including the one individual followed up till 30 yrs post infection (Fig. 1a,b). Anti-HEV antibody positivity in the studied individuals at different time points following recovery from HEV infection are as follows: 1/1 at 1 year post HEV infection, 26/29 at 3 years, 1/1 at 4 years, 1/1 at 8 years, 2/2 at 16 years and 1/1 at 30 years post infection (Fig. 1b). IgG avidity indices of 30/31 acute hepatitis E patients were low (<40%) whereas 1/31 was marginally equivocal indicating primary infection, while those in all hepatitis E recovered individuals were high indicating previous exposure (Fig. 1c).

### Induction of T cells and subsets in hepatitis E recovered individuals upon stimulation with recombinant HEV capsid protein (rORF2p)

**Figure 2:**
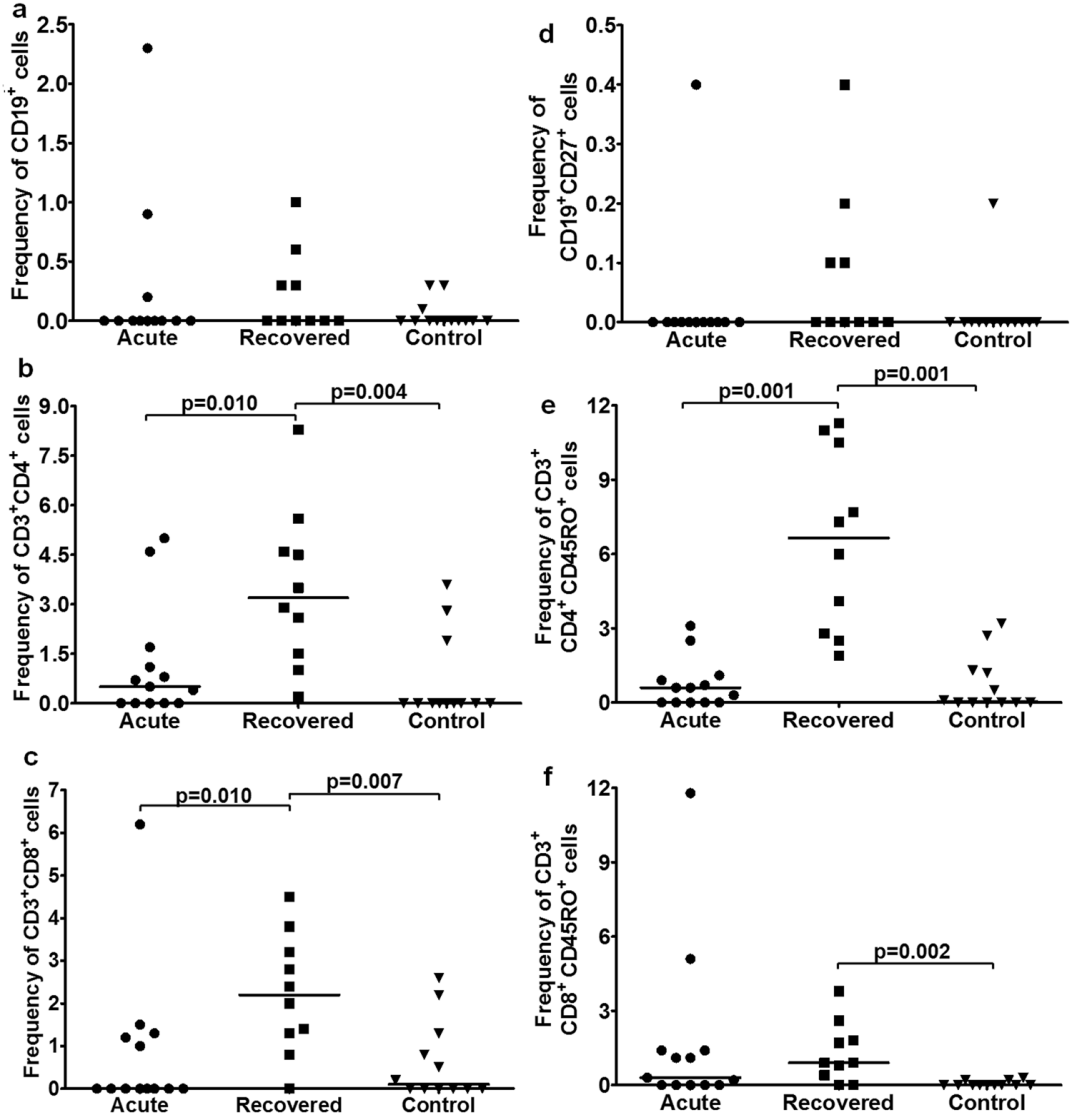
Flow cytometric analysis of B, T, memory B and T cells upon stimulation with recombinant open reading frame 2 protein (rORF2p). Peripheral blood mononuclear cells (PBMCs) from 13 acute hepatitis E patients, 10 hepatitis E recovered individuals, and 13 healthy controls were cultured in the presence/absence of rORF2p. After 72 hrs, cells were harvested, stained and acquired on flow cytometer. Frequencies of rORF2p-stimulated (**a**) B, (**b**) T_H_, (**c**) T_C_, (**d**) memory B, (**e**) memory T_H_ and (**f**) memory T_C_ cells were determined after normalization with unstimulated cells. Data are presented as percentage of immune cells out of lymphocytes. The dots represent individual values and bars represent median values.

**Figure 3:**
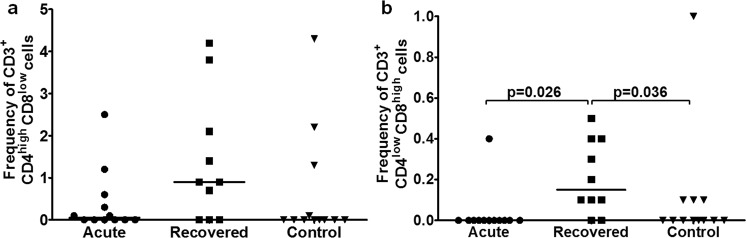
Flow cytometric analysis of rORF2p-stimulated double positive T (DPT) cells. Unstimulated/rORF2p-stimulated PBMCs from 13 acute hepatitis E patients, 10 hepatitis E recovered individuals and 13 healthy controls were stained and frequencies of rORF2p-stimulated (**a**) CD3^+^ CD4^high^ CD8^low^ and (**b**) CD3^+^ CD4^low^ CD8^high^ cells were determined after normalization with unstimulated cells. Data are presented as percentage of immune cells out of lymphocytes. The dots represent individual values and bars represent median values.

Frequencies of immune cells expressed as median (range) are as follows:

*B cells:* Frequency was comparable among all study groups [acute: 0 (0–2.3), recovered: 0 (0–1), controls: 0 (0–0.3)] (Fig. 2a). *T cells:* T_H_ cell frequency was higher in hepatitis E recovered individuals compared to acute hepatitis E patients and healthy controls [recovered: 3.2 (0.2–8.3) vs. acute: 0.5 (0–5), p = 0.010; recovered vs. controls: 0 (0–3.6), p = 0.004] (Fig. 2b). T_C_ cell frequency was higher in hepatitis E recovered individuals compared to acute hepatitis E patients and healthy controls [recovered: 2.2 (0–4.5) vs. acute: 0 (0–6.2), p = 0.010; recovered vs. controls: 0.1 (0–2.6), p = 0.007] (Fig. 2c). *Memory B cells:* Frequency was comparable among all groups [acute: 0 (0–0.4), recovered: 0 (0–0.4), controls: 0 (0–0.2)] (Fig. 2d). *Memory T cells:* Memory T_H_ cell frequency was higher in hepatitis E recovered individuals compared to acute hepatitis E patients and healthy controls [recovered: 6.65 (1.9–11.3) vs. acute: 0.6 (0–3.1), p = 0.001; recovered vs. controls: 0.05 (0–3.2), p = 0.001] (Fig. 2e). Memory T_C_ cell frequency was higher in hepatitis E recovered individuals compared to healthy controls [recovered vs. controls: 0 (0–0.3); p = 0.002] (Fig. 2f). *Double positive T (DPT) cells:* CD3^+^ CD4^high^ CD8^low^ cell frequency was comparable among all groups [acute: 0.05 (0–2.5), recovered: 0.9 (0–4.2), controls: 0 (0–4.3)] (Fig. 3a). CD3^+^ CD4^low^CD8^high^ cell frequency was higher in hepatitis E recovered individuals compared to acute hepatitis E patients and healthy controls [recovered: 0.15 (0–0.5) vs. acute: 0 (0–0.4), p = 0.026; recovered vs. controls: 0 (0–1), p = 0.036] (Fig. 3b).

### Functional memory B cells 1–16 years post HEV infection

**Figure 4:**
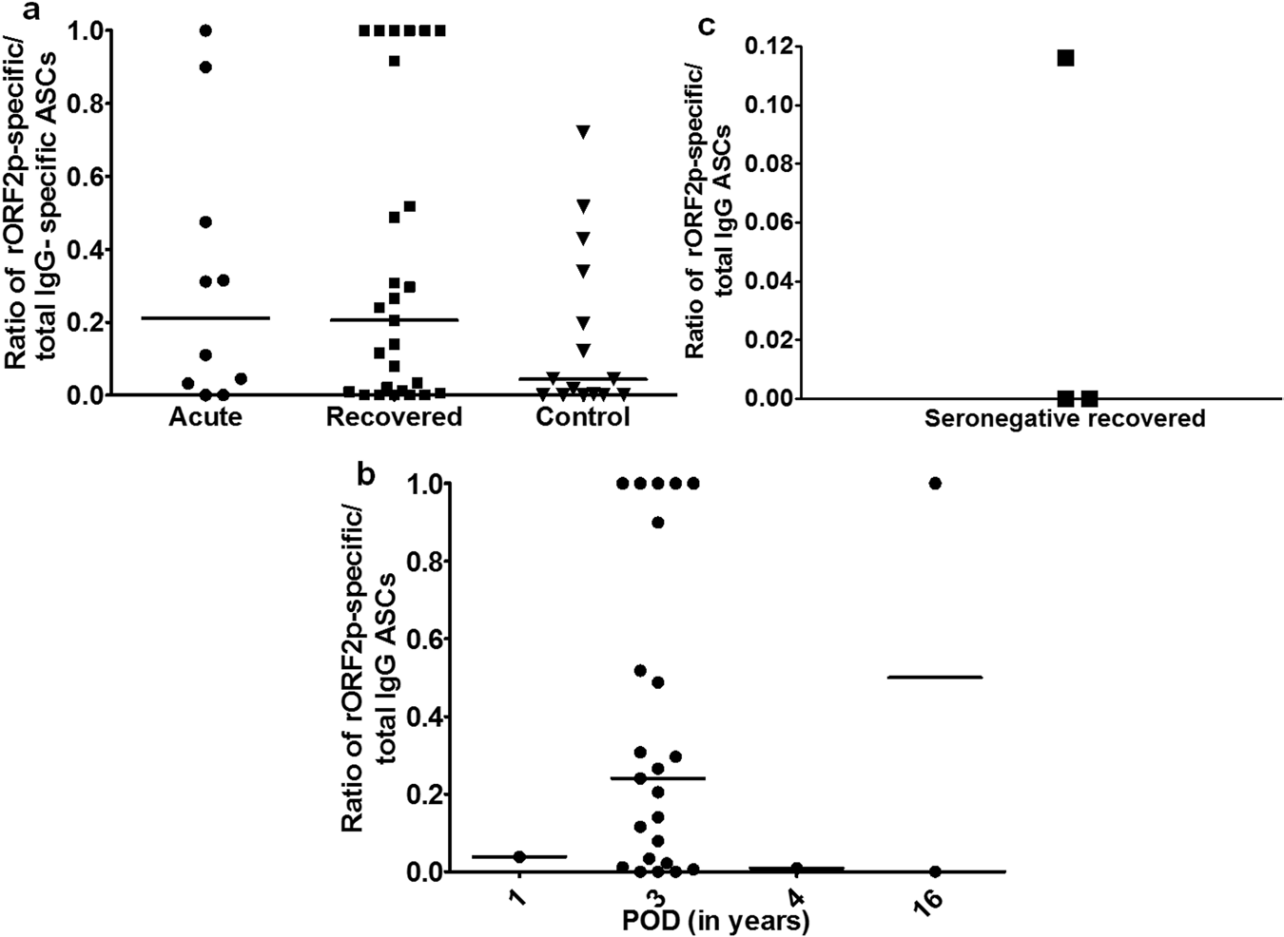
Memory B cell responses by ELISPOT assay. Total IgG and HEV-specific antibody secreting cells (ASCs) per million PBMCs were enumerated by memory B cell ELISPOT assay in 10 acute hepatitis E patients, 27 hepatitis E recovered individuals and 15 healthy controls. The dots represent individual values and bars represent median values. The observed ratio of HEV-specific and total IgG ASCs is shown (**a**) among all study groups, (**b**) in seropositive hepatitis E recovered individuals 1–30 years post infection and (**c**) in seronegative hepatitis E recovered individuals.

The ratios of HEV-specific antibody secreting cells (ASCs) and total IgG ASCs were comparable among all the study groups [Median (range) are acute: 0.2 (0–1), recovered: 0.2 (0–1), controls: 0.04 (0–0.7)] (Fig. 4a). In recovered group, memory B cells were functional in ~95% of anti-HEV positive subjects (n = 24) 1–16 years post infection (Fig. 4b) and in 33% of anti-HEV negative subjects (n = 3) (Fig. 4c). Unfortunately, it was not possible to study functionality of memory B cells in the recovered individual at 30 years post infection.

### Absence of cytokine response by memory T cells in recovered individuals

**Figure 5:**
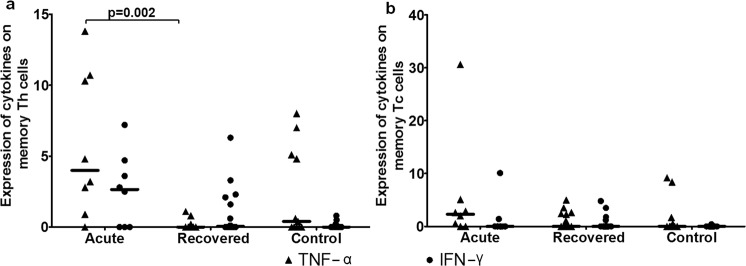
Intracellular cytokine staining for TNF-α and IFN-γ. Expressions of TNF-α and IFN-γ on (**a**) memory T_H_ and (**b**) memory T_C_ cells were determined in 8 acute hepatitis E patients, 16 hepatitis E recovered individuals and 11 healthy controls. The dots represent individual values and bars represent median values.

TNF-α expression on memory T_C_ cells and IFN-γ expression on memory T_H_ and T_C_ cells were comparable among all studied groups (Fig. 5a,b). TNF-α expression was higher on memory T_H_ cells of acute hepatitis E patients compared to hepatitis E recovered individuals [Median (range): acute: 4 (0–13.8) vs. recovered: 0 (0–1.1), p = 0.002] (Fig. 5a). The frequencies of TNF-α^+^ and IFN-γ^+^ memory T_H_ cells in positive controls were 15.5 (0.5–60.5) and 10.4 (0.5–46.4) respectively, and those of TNF-α^+^ and IFN-γ^+^ memory T_C_ cells were 16.8 (0.2–46.6) and 14.2 (0.4–66.5) respectively.

### Higher frequencies of effector (T_EM_) and central (T_CM_) memory T cells in recovered individuals

**Figure 6:**
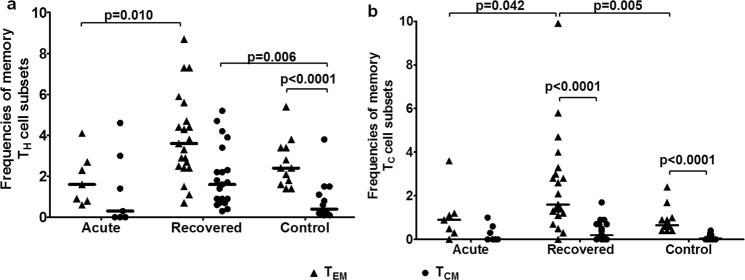
Frequencies of effector (T_EM_) and central (T_CM_) memory T cells. PBMCs from 7 acute hepatitis E patients, 21 hepatitis E recovered individuals and 12 healthy controls were used to assess the frequencies of T_CM_ (CCR7^+^ CD62L^+^) and T_EM_ (CCR7^−^ CD62L^−^) subsets of (**a**) CD3^+^ CD4^+^ CD45RO^+^ memory T_H_ and (**b**) CD3^+^ CD8^+^ CD45RO^+^ memory T_C_ cells. The dots represent individual values and bars represent median values.

Among the patient categories, CD4^+^ T_EM_ cells were higher in hepatitis E recovered individuals compared to acute hepatitis E patients [Median (range): acute: 1.6 (0.6–4.1) vs. recovered: 3.6 (0.7–8.7), p = 0.010] (Fig. 6a). CD4^+^ T_CM_ cells were higher in hepatitis E recovered individuals compared to healthy controls [recovered: 1.6 (0.3–5.2) vs. controls 0.4 (0.1–3.8), p = 0.006] (Fig. 6a). CD8^+^ T_EM_ cells were higher in hepatitis E recovered individuals compared to acute hepatitis E patients and healthy controls [recovered: 1.6 (0–9.9) vs. acute: 0.9 (0–3.6), p = 0.042; recovered vs. controls: 0.65 (0.4–2.4), p = 0.005] (Fig. 6b).

### Lack of correlation between serological memory and memory B cell response in hepatitis E recovered individuals

There was no correlation between plasma anti-HEV IgG levels and ratio of HEV-specific ASCs and total IgG ASCs as assessed by spearman correlation analysis (data not shown).

## Discussion

HEV infection is a global health challenge; yet pressing issues pertaining to longevity of immunological memory and protective immunity against HEV have remained partly answered. Till date, the generation and persistence of anti-HEV antibodies are considered synonymous with the development of natural as well as vaccine-induced immunity. However, studies to correlate B and T cell responses to hepatitis E vaccine efficacy have not been undertaken. Additional correlates of protection (CoPs) against HEV infection may facilitate the assessment of the efficacy of future hepatitis E vaccine candidates.

Ninety one percent of the studied hepatitis E recovered individuals remained anti-HEV positive including the one at 30 years post HEV infection, indicating persistence of serological memory maintained by long-lived plasma cells^16^. A study from our lab had reported anti-HEV IgG titers as low as 1:100 to be protective against the development of clinical hepatitis E^17^. Majority of currently studied hepatitis E recovered individuals including the one at 30 years post primary exposure had anti-HEV antibodies that could be associated with lower likelihood to develop symptomatic infection. However, during a hepatitis E outbreak, passive administration of immune serum globulins to pregnant women didn’t impart protection^17^ indicating that antibodies could just be a surrogate and may be other immune cells prevent symptomatic infection.

India is hyperendemic for hepatitis E, and subclinical HEV infections are common^18^. The low anti-HEV IgG avidity indices of the studied acute hepatitis E patients indicated that these were the cases of primary exposure, and not of re-infection. IgG avidity assay can be employed for assessing whether hepatitis E vaccine candidate elicits long term protective immunity as was reported in case of measles vaccine^19^, especially in case of vaccinated individuals who become seronegative overtime. Generation of low avidity antibodies upon HEV re-exposure in these individuals will clearly indicate the vaccine failure.

Immunological memory (both B and T cell), a cardinal feature of the adaptive immune system, is the cornerstone of protective immunity. In case of parvovirus and HBV infections, memory B cells were shown to be correlated with protection even when the antibody titers were low or undetectable^20,21^. We had highlighted the utility of memory B cell recall responses in assessing the immunogenicity of hepatitis E vaccine candidate in mice^22^. HEV rORF2p-specific ASCs in 95% of recovered seropositive individuals 1–16 years post HEV infection established the presence of functional memory B cells, regardless of the observed decline in antibody levels. In hepatitis B vaccinated individuals, memory B cell recall responses were observed even after the loss of antibodies^23^. In our study, when memory B cell response was assessed in HEV seronegative recovered individuals (3 years post infection), HEV rORF2p-specific anamnestic response was observed in 33%. Despite very small sample size, this observation is significant indicating that in the absence of circulating anti-HEV IgG antibodies, HEV-specific memory B cells may differentiate into plasma cells upon re-exposure to HEV antigen. This also raises an intriguing question: whether antibodies should be the only CoPs against HEV infection? The lack of correlation between memory B cell frequency, anti-HEV IgG antibody levels and HEV rORF2p-specific ASCs goes in parallel with a study in hepatitis B vaccinees^24^.

Presence of functional memory B cells in HEV naïve control individuals is surprising though a similar situation of positive B cell response was observed in HEV seronegative residents, indicating prior subclinical HEV infection^25^.

Despite higher frequencies of rORF2p-stimulated memory T cells, lack of effector functionality of memory T cells in terms of cytokine response in the studied hepatitis E recovered individuals could be suggestive of waning of memory T cell response overtime. Similar observation of insufficient cytokine responses in recovered individuals 2 months post HEV infection has been reported by our group^26^. TNF-α expressing CD3^+^ CD4^+^ CD45RO^+^ T cells in acute hepatitis E patients may represent the primed effector T cells that transiently express CD45RO^27^.

Observed HEV rORF2p-stimulatedCD3^+^ CD4^low^ CD8^high^ cells in hepatitis E recovered individuals could be the effector memory T cells, since DPT cells have been reported to be antigen-specific effector memory cells^28,29^. Central memory is most useful in diseases with long incubation periods, but effector memory is critical to protection against most infections^30^. Thus, higher percentage of CD4^+^ T_EM_ and T_CM_ cells and CD8^+^ T_EM_ cells in the hepatitis E recovered individuals is an important observation.

Antibodies and memory B cells are CoPs against HBV infection^21,31^. Though antibodies are considered as correlates of protection for assessing the efficacy of hepatitis E vaccine candidates, anti HEV antibodies is not the whole story as evident from the reports of breakthrough infections in seropositive vaccinated individuals^32^, hence multiple correlates of protective immunity against HEV are needed for the assessment of the degree of protection conferred by hepatitis E vaccine candidates. CoPs are derived from vaccine efficacy data, nevertheless our data on hepatitis E recovered individuals indicate towards serum anti-HEV antibodies and circulating functional memory B cells (ASCs), both as potential co-correlates of protection. Keeping these things in mind, optimal strategies should be designed to assess HEV-vaccine efficiency.

In conclusion, HEV-specific humoral responses are stably maintained for several years in most hepatitis E recovered individuals. In addition to anti-HEV antibodies, possible role of memory B cell response against HEV re-infection could also be considered. These data may have an implication in the assessment of HEV vaccine.

## Methods

### Antigen preparation

Complete open reading frame 2 (ORF2) (1983 bp: 5147–7129 nt), encoding HEV capsid protein, from HEV genotype 1 was cloned in pFastBac1 vector earlier in our lab. Using this construct, rORF2p was expressed in Sf9 insect cell line and purified by anion exchange high performance liquid chromatography using AKTA BASIC 100 system (Amersham Biosciences, UK)^33^.

### Study groups

Three groups of subjects were included; i.e., a cohort (n = 95) of acute hepatitis E patients (n = 31), hepatitis E recovered individuals (n = 35) and healthy controls (n = 29). All human infections in India are caused by HEV genotype 1^34^.

#### Inclusion criteria

Acute hepatitis E patients: The patients had icterus, dark-colored urine, elevated alanine aminotransferase (ALT) (normal level, 4–40 IU/L) and/or bilirubin levels (>1 mg/ml) in the plasma and/or presence of bile salts and pigments in the urine, and were positive for anti-HEV IgM and IgG antibodies. These standard clinical and biochemical criteria were used for the classification of patients as acute hepatitis E patients^35^. The acute patients were from different outbreaks in India. *Hepatitis E recovered individuals:* With a previous history of clinical hepatitis E, were negative for anti-HEV IgM and positive (n = 32) or negative (n = 3) for anti-HEV IgG antibodies with normalized ALT levels. The recovered individuals were known to have acute hepatitis E in the past from previously investigated hepatitis E outbreaks. The incidence of HEV re-infection was ruled out by the absence of clinical hepatitis E in these individuals after recovery from primary infection. *Healthy controls:* Without a history of clinical hepatitis E, were negative for anti-HEV IgM/IgG with normal ALT levels and were of the similar age as the patient groups and were from the same region as that of the outbreaks. They were collected at the same time as the other two groups. Written informed consent was taken from all healthy controls. The details of all the outbreaks are given in Table 2.

**Table 2:** Details of the outbreaks. Data are shown as median (range); NA: Not applicable.

Outbreak location	Year	Post onset days of illness (POD) (Days)	No. of participants	Age	Sex ratio (Male:Female)
**Acute hepatitis E patients**					
Satara, Maharashtra	2014	7 (2–17)	13	36 (17–64)	7:6
Shimla, Himachal Pradesh	2016	4 (3–7)	10	28 (15–56)	7:3
Pune, Maharashtra	2016	8 (7–12)	8	22 (11:36)	3:1
**Outbreak location**	**Year**	**POD (Years)**	**No. of participants**	**Age**	**Sex ratio (Male:Female)/Sex**
**Hepatitis E recovered individuals**					
Bhor, Maharashtra	2013	3	29	38 (12–65)	12:17
Pune, Maharashtra	2015	1	1	39	Male
	2012	4	1	60	Male
	2008	8	1	33	Male
	2000	16	2	52 (45–58)	Males
	1986	30	1	80	Male
**Outbreak location**	**Year**	**POD**	**No. of participants**	**Age**	**Sex ratio (Male:Female)**
**Healthy controls**					
Satara, Maharashtra	2014	NA	5	29 (23–35)	3:2
Shimla, Himachal Pradesh	2016	NA	4	25 (22–44)	2:2
Bhor, Maharashtra	2013	NA	11	30 (21–56)	6:5
Pune, Maharashtra	2015	NA	10	28 (22–58)	5:4

#### Exclusion criteria

Individuals with other viral infections including HBV, HCV and human immunodeficiency virus infections were excluded.

The study was approved by the “Institutional Ethics Committee for Research on Humans” as per the guidelines of Indian Council of Medical Research, New Delhi, India. Written informed consent was obtained from all the study participants in accordance with the Declaration of Helsinki. Guardian’s consent was obtained when the participants were minor. Blood sample was drawn from each study participant; plasma was used for serological and biochemical assays, while peripheral blood mononuclear cells (PBMCs) were used for the assessment of B and T cell responses. Due to ethical issues, small volumes of blood samples were drawn from the study participants resulting in limited availability of PBMCs. Hence, all samples were not processed for all the assays leading to variable sample size per assay. The numbers of samples tested for each assay are shown in Supplementary Fig. S1. Assays were performed only when the viability of cells was >98% as observed by Trypan blue staining.

### Serological and biochemical assays

#### Determination of ALT levels

The plasma samples of all study participants were tested for ALT levels using commercial kit (Span Diagnostics, India) as per the manufacturer’s instructions. ALT levels >40 IU/ml were considered as elevated.

#### Detection of anti-HEV IgM and IgG antibodies

The detection of anti-HEV IgM and IgG antibodies was done using Wantai HEV IgM/IgG ELISA kits according to the manufacturer’s instructions (Beijing Wantai Biological Pharmacy Enterprise Co. Ltd., China)

#### Determination of IgG avidity index

Antibodies generated upon primary antigen exposure exhibit low avidity, whereas those with high avidity are generated upon secondary exposure to antigen due to affinity maturation. In order to ensure that all acute hepatitis E patients are cases of primary HEV infection, plasma samples from acute hepatitis E patients and hepatitis E recovered individuals were used to assess IgG avidity using Wantai HEV IgG ELISA kits (Beijing Wantai Biological Pharmacy Enterprise Co. Ltd., China) as described previously^36^. IgG avidity index (%) was calculated as: (O.D. of well with urea/O.D. of well without urea) ×100. An index <40% was considered as low, >60% as high and 40–60% as equivocal.

### Frequencies of rORF2p-stimulated B, T, memory B and T cells

PBMCs isolated from blood samples of 13 acute hepatitis E patients, 10 hepatitis E recovered individuals and 13 healthy controls were cultured in Roswell Park Memorial Institute (RPMI) 1640 medium with/without rORF2p for 72 hrs. Cells were harvested and 0.2 × 10^6^ unstimulated/stimulated cells were surface stained using pre-titrated anti-human CD19-PECy7, CD27-APCH7, CD3-FITC, CD4-PE, CD8-PerCpCy5.5 and CD45RO-APC antibodies (BD Biosciences, CA, USA). For antibody titration, same number of cells from the same sample was stained with different concentrations of the antibodies. After acquisition and analysis, a point of saturation was reached where 2 consecutive concentrations yielded same results. The lower concentration of antibody was used for staining all the samples. For each sample, 50,000 events were acquired in BD FACS Aria-II flow cytometer and analyzed using BD FACS Diva software (BD Biosciences, CA, USA). The strategy for gating B, T, memory B and T cells is depicted in Supplementary Fig. S2. B (CD19^+^), memory B (CD19^+^ CD27^+^), T_H_ (CD3^+^ CD4^+^) and T_C_ (CD3^+^CD8^+^) cells were gated from lymphocytes and memory T_H_ (CD3^+^ CD4^+^ CD45RO^+^) and T_C_ (CD3^+^ CD8^+^ CD45RO^+^) cells were gated from their parent cells. DPT cells were defined as CD3^+^ CD4^high^ CD8^low^ and CD3^+^ CD4^low^ CD8^high^ cells. Data from stimulated cells were analyzed after normalization with that of unstimulated cells for each sample. The representative plots for unstimulated and stimulated cells from a sample are shown in Supplementary Fig. 4.

### Memory B cell functional assay

Memory B cells activate and differentiate into ASCs in response to antigen or upon polyclonal stimulation. B cell ELISPOT assay was performed to assess the HEV-specific ASCs generated from memory B cells as shown in Supplementary Fig. S3. Briefly, 1.5 × 10^6^ PBMCs from 10 acute hepatitis E patients, 27 hepatitis E recovered individuals and 15 healthy controls were cultured in the presence/absence of pokeweed mitogen (100 ng/ml; Sigma, St Louis, MO, USA) and *Staphylococcus aureus* Cowan strain (1:20,000; Sigma, St Louis, MO, USA) for 3 days. On day 2, 96-well Elispot plates (Millipore Bedford, MA, USA) were coated with 20 µg/ml rORFp or 5 µg/ml anti-IgG antibody (Mabtech, Sweden) at 4 °C overnight. On day 3, the plates were washed with PBS containing 0.5% (v/v) Tween-20 and blocked with RPMI-1640 + 10%FBS at 37 °C for 3–4 h. PBMCs were harvested and 1 × 10^5^ unstimulated/stimulated cells per well were added in triplicates to rORF2p-coated wells, while 1 × 10^5^ stimulated cells per well were added to anti-IgG-coated wells, along with anti-HEV IgG-biotinylated detection antibody (1:100; Mabtech, Sweden) and incubated at 37 °C. After 24 hrs, the plate was developed using 3-amino-9-ethylcarbazole substrate as described previously^37^ and spots were counted on ELISPOT reader (AID, Strassberg, Germany). The number of spots observed in rORF2p-coated wells with stimulated cells was normalized with those observed with unstimulated cells for each sample to determine the number of HEV-specific ASCs. The results are expressed as ratio of HEV-specific ASCs/total IgG ASCs per million PBMCs. A result was considered as negative when no rORF2p-specific ASCs were observed.

### Cytokine response by memory T cells

Based on the fact that IFN-γ and TNF-α are the predominant cytokines produced by T cells when activated, the functionality of HEV-specific memory T cells was assessed by intracellular cytokine staining. 0.3 × 10^6^ PBMCs from 8 acute hepatitis E patients, 16 hepatitis E recovered individuals and 11 healthy controls were cultured in the presence of anti-CD3 and anti-CD28 (0.5 µg/ml; Miltenyi Biotec, Germany), with/without 10 µg/ml rORF2p for 6 hrs, with addition of Brefeldin A (Sigma, USA) 2 hrs post stimulation. The cells were harvested and surface stained for memory T cell markers, fixed and permeabilized with 200 µl BD cytofix/cytoperm buffer (BD Biosciences, USA), and processed for intracellular staining with TNF-α-PECy7 and IFN-γ-BV510 antibodies as per manufacturer’s instructions (BD Biosciences, CA, USA). For each sample, 50,000 events were acquired in BD FACS Aria-II flow cytometer using BD FACS Diva software (BD Biosciences, CA, USA). Data from stimulated cells were analyzed after normalization with unstimulated cells. For positive control, PBMCs were stimulated with phorbol 12-myristate 13-acetate (0.05 µg/ml) and ionomycin (1 µg/ml) (Sigma, MO, USA).

### Frequencies of memory T cell subsets

Both effector and central memory T cells persist for years and may play key roles toward protection against infection. Freshly isolated PBMCs (0.2 × 10^6^) from 7 acute hepatitis E patients, 21 hepatitis E recovered individuals and 12 healthy controls were surface stained for memory T cell markers along with anti-human CCR7- PECy7 and CD62L-V450 antibodies (BD Biosciences, CA, USA). For each sample, 50,000 events were acquired in BD FACS Aria-II flow cytometer. The phenotypes of T_CM_ and T_EM_ cells were CD3^+^ CD4/8^+^ CD45RO^+^ CCR7^+^ CD62L^+^ and CD3^+^ CD4/8^+^ CD45RO^+^ CCR7^−^ CD62L^−^ respectively.

### Statistics

All the statistical analyses were performed using SPSS 20 software (SPSS Inc., IL, USA). For intergroup comparisons, nonparametric Mann–Whitney U-test (where difference in variances <4) or Kolmogorov–Smirnov test (where difference in variances >4) were used. Correlation among anti-HEV IgG levels and ratio of HEV-specific ASCs/total IgG ASCs was assessed using Spearman’s rank correlation. All the data are expressed as median (range). A p value of <0.05 is considered significant.

## Supplementary information

Supplementary information
