# Supplementary material for: Antibody and Memory B Cell Responses in Hepatitis E Recovered Individuals, 1–30 Years Post Hepatitis E Virus Infection
Source: Sci Rep. 2019 Mar 11;9:4090. doi: 10.1038/s41598-019-40603-9 (PMC6411774; doi:10.1038/s41598-019-40603-9)

**Antibody and Memory B Cell Responses in Hepatitis E Recovered Individuals, 1-30 Years Post Hepatitis E Virus Infection**

Shruti P. Kulkarni, Meenal Sharma, Anuradha S. Tripathy

**Supplementary figure S1:** Flow chart showing the number of samples tested in all the assays.

**
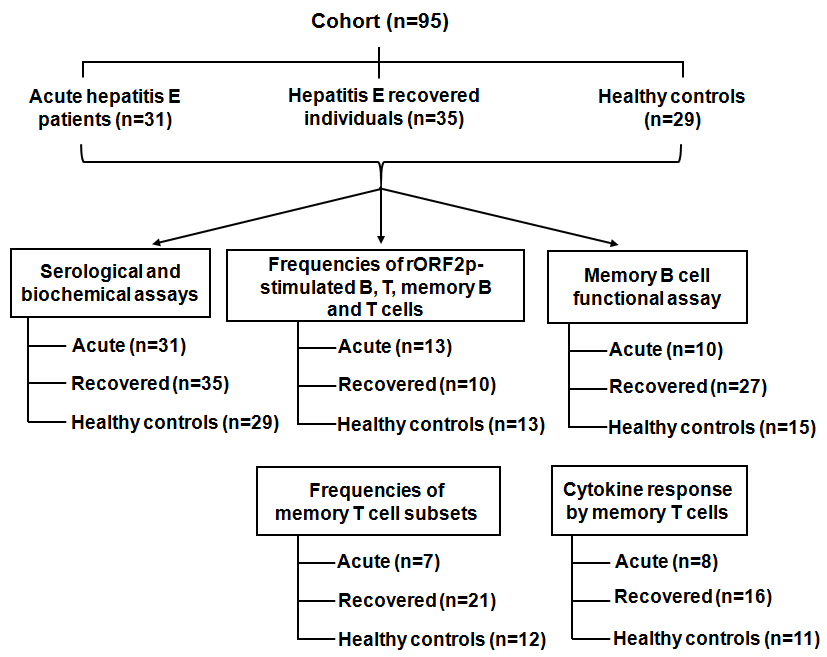
**

**Supplementary figure S2:** Plots showing the strategy used for gating B, T, memory B and T cells. Lymphocytes were gated from forward vs. side scatter dot plot. B (CD19^+^), memory B (CD19^+^CD27^+^), T_H_ (CD3^+^CD4^+^) and T_C_ (CD3^+^CD8^+^) cells were gated from lymphocytes, and memory T_H_ (CD3^+^CD4^+^CD45RO^+^) and T_C_ (CD3^+^CD8^+^CD45RO^+^) cells were gated from T_H_ and T_C_ cells respectively.

**
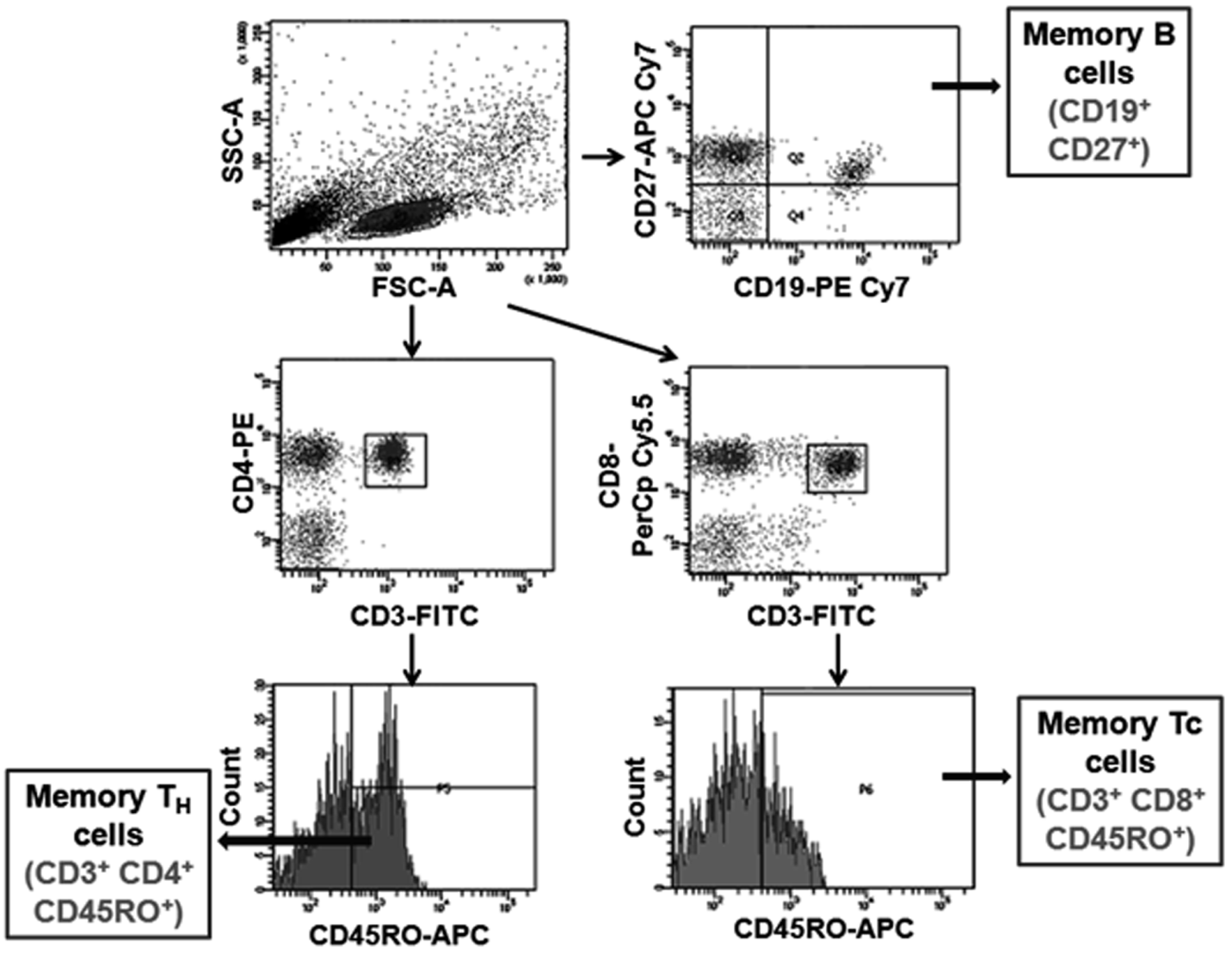
**

**Supplementary figure S3:** Flow chart depicting the memory B cell ELISPOT protocol for assessing the functionality of HEV-specific memory B cells.


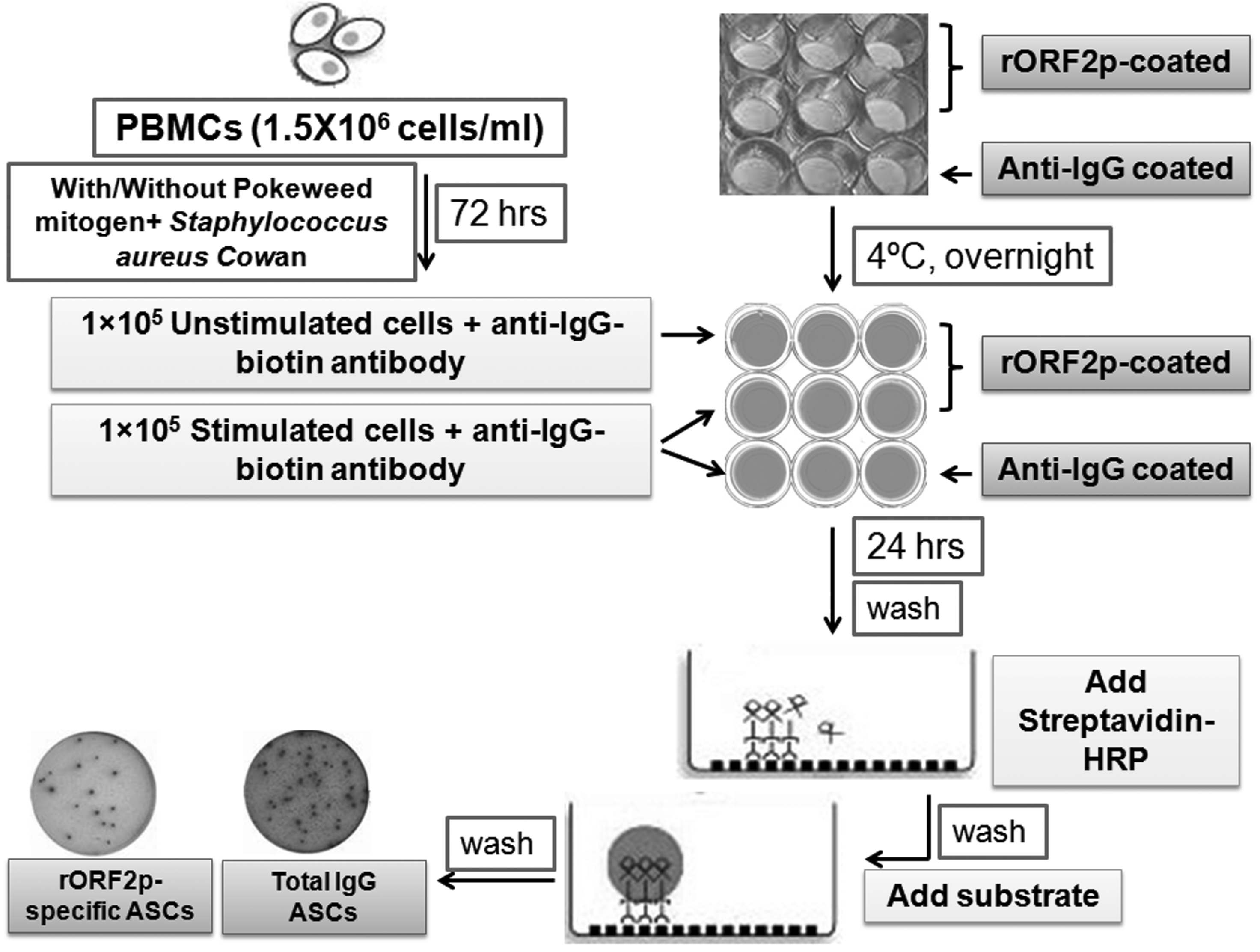


**Supplementary figure S4:** Representative plots of (A) unstimulated and (B) stimulated cells of a sample for intracellular cytokine staining.

(A)


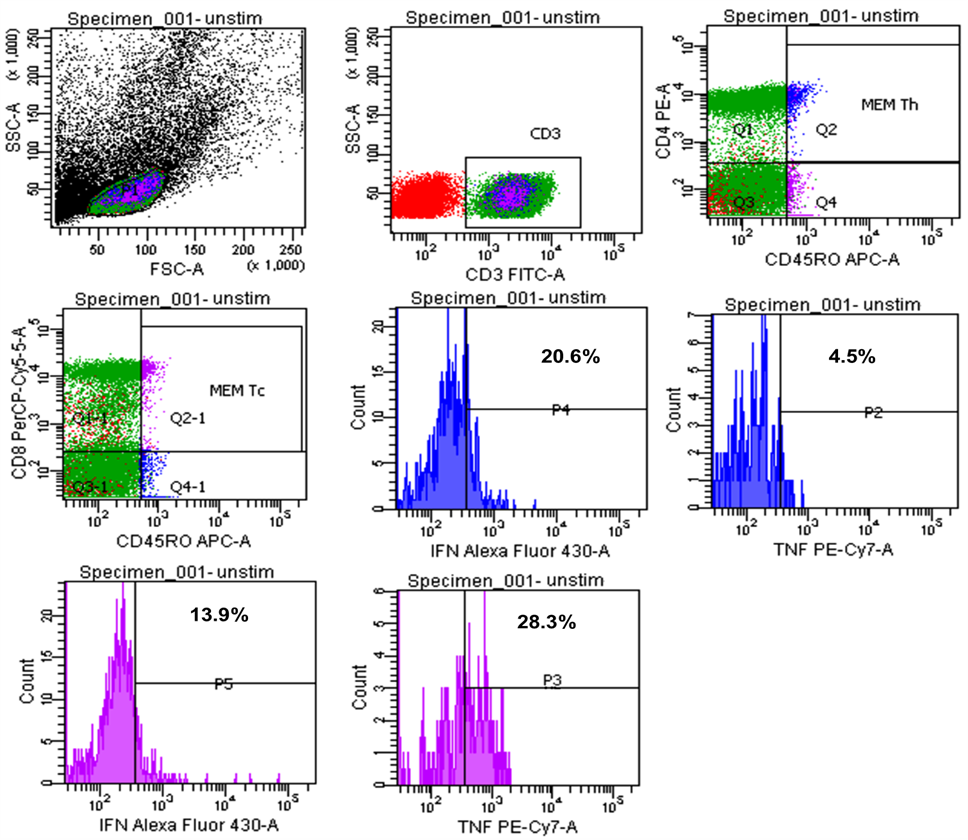


(B)


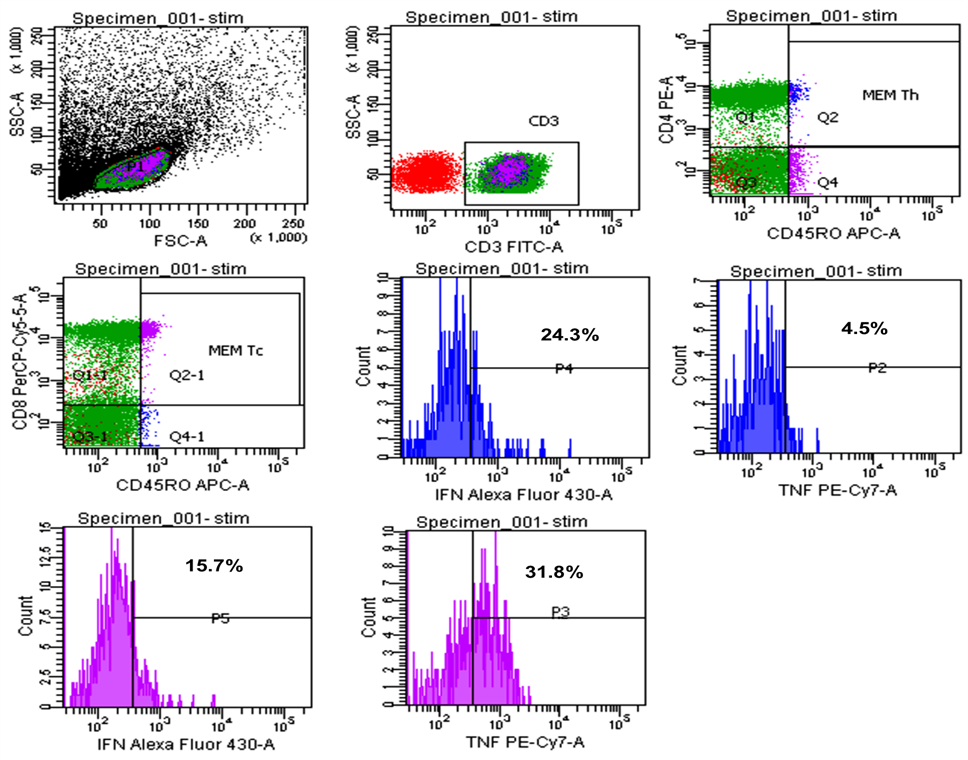

Supplement: Supplementary file 1 — Supplementary information [file 41598_2019_40603_MOESM1_ESM.doc]
